# Clinical systematic reviews – a brief overview

**DOI:** 10.1186/s12874-023-02047-8

**Published:** 2023-10-10

**Authors:** Mayura Thilanka Iddagoda, Leon Flicker

**Affiliations:** 1 University of Western Australia, Stirling Hwy, Crawley, Perth, WA 6009 Australia; 2 Perioperative Service, Royal Perth Hospital, Wellington Street, Perth, WA 6000 Australia

**Keywords:** Sytematic review, Meta-analysis, Effect measure, Heterogeneity, Risk of bias, Certainty of evidence

## Abstract

**Objective:**

Systematic reviews answer research questions through a defined methodology. It is a complex task and multiple articles need to be referred to acquire wide range of required knowledge to conduct a systematic review. The aim of this article is to bring the process into a single paper.

**Method:**

The statistical concepts and sequence of steps to conduct a systematic review or a meta-analysis are examined by authors.

**Results:**

The process of conducting a clinical systematic review is described in seven manageable steps in this article. Each step is explained with examples to understand the method evidently.

**Conclusion:**

A complex process of conducting a systematic review is presented simply in a single article.

Systematic reviews are a structured approach to answer a research question based on all suitable available empirical evidence. The statistical methodology used to synthesize results in such a review is called ‘meta-analysis’. There are five types of clinical systematic reviews described in this article (see Fig. 1), including intervention, diagnostic test accuracy, prognostic, methodological and qualitative. This review will provide a very brief overview in a narrative fashion. This article does not cover systematic reviews of more epidemiologically based studies. The recommended process undertaken in a systematic review is described under seven steps in this paper [1].

**Fig. 1:**
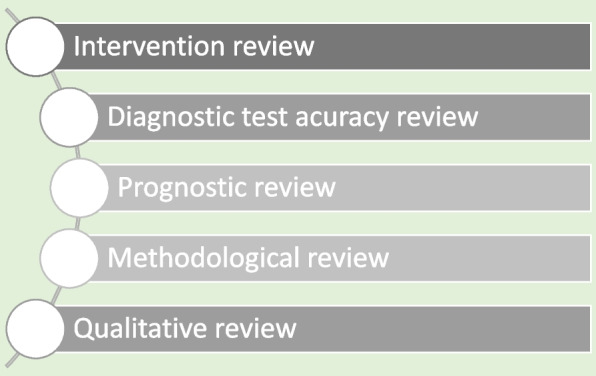
Types of systematic reviews

There are resources for those who are moving from the beginning stage and gaining more expertise (See Table 1). Cochrane conducts online interactive master classes on systematic reviews throughout the year and there are web tutorials in the form of e-learning modules. Some groups in Cochrane commission limited number of systematic reviews and can be contacted directly for support (contact@cochraneresponse.com). Some institutions have systematic review training programs including John Hopkins (Coursea), Joanna Briggs Institute (JBI education), Yale University (Search strategy), University of York (Centre for Reviews) and Mayo Clinic Libraries. BMC systematic reviews group also introduced “Peer review mentoring” program to support early researchers in systematic reviews. The local University/Hospital librarian is usually a good point of first reference for searches and is able to direct reviewers to other support.

**Table 1:** Recourses and training for systematic reviews and meta-analysis

Institue	Training program	Link
Cochrane	Cochrane interactive learning modules on conducting systematic reviews	https://training.cochrane.org/interactivelearning
	Cocrane Guide and handbooks on systematic review and meta-analysis	https://training.cochrane.org/handbooks
John Hopkins Institute	Introduction to Systematic Review and Meta-Analysis (Coursera)	https://www.coursera.org/learn/systematic-review
Joanna Briggs Institute (JBI)	Comprehensive Systematic Review Training Program	https://jbi.global/education/systematic-review-training
University of York—Centre for Reviews and Dissemination	Introduction to Systematic Reviews and Critical Appraisal Course	https://www.york.ac.uk/crd/training-services/introduction-to-systematic-reviews/
Mayo Clinc Library	Systematic Reviews: Training Resources	https://libraryguides.mayo.edu/systematicreviewprocess/training
University of Toronto	Online Courses on Systematic Reviews	https://guides.hsict.library.utoronto.ca/c.php?g=430254&p=5066235

## Research question and study protocol

A clearly defined study question is vital and will direct the following steps in a systematic review. The question should have some novelty (e.g. there should be no existing review without new primary studies) and be of interest to the reviewers. Major conflicts of interest can be problematic (e.g. employment by a company that manufactures the intervention). Primary components of a research question should include inclusion criteria, search strategy, analysis or outcome measures and interpretation. Types of reviews will determine the categories of research questions such as intervention, prognostic, diagnostic, etc. [1].

**Fig. 2:**
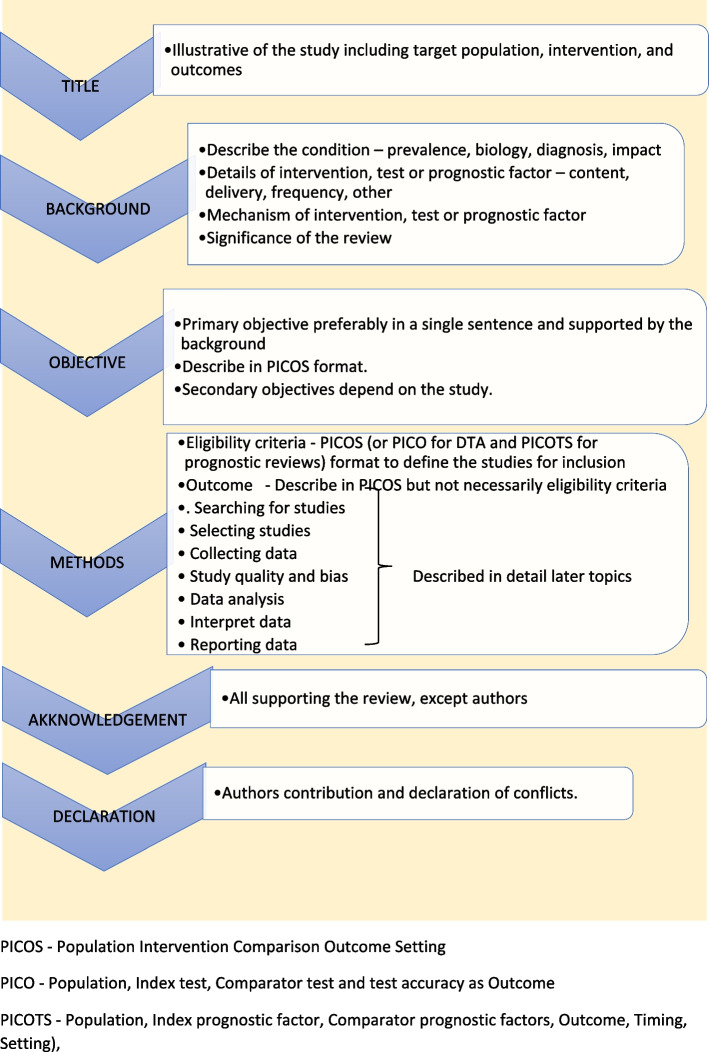
Structure of the review protocol

Study protocol elaborates the research question. The language of the study protocol is important. It is usually written in future tense, accessible language, active voice and full sentences [2]. Structure of the review protocol is described in Fig. 2.

## Searching studies

The comprehensive search for eligible studies is the most defining step in a systematic review. The guidance by an information specialist, or an experienced librarian, is a key requirement for designing a thorough search strategy [3, 4].

### Planning

The search strategy should explore multiple sources rigorously and it should be reproducible. It is important to balance sensitivity and precision in designing a search plan. A sensitive approach will provide a large number of studies, which lowers the risk of missing relevant studies but may produce a large workload. On the other hand, a focused search (precision) will give a more manageable number of studies but increases the risk of missing studies.

There are multiple sources to search for eligible studies in a systematic review or a meta-analysis. The key databases are Central (Cochrane register of clinical trials), MEDLINE (PubMed) and Embase. There are many other databases, published reviews and reference lists that may be used. Forward citation tracking can be done for searched studies using citation indices like Google Scholar, Scopus or Web of Science. There may be studies presented to different levels of governmental and non-governmental organizations which are not recognized as commercial publishers. These studies are called ‘grey literature’. Extensive investigations in different sources are required to identify grey literature. Information specialists are helpful in finding these studies [2].

### Designing

Designing the search strategy requires a structured approach. Again, assistance from a librarian or an information specialist is recommended. PICOS, PICO and PICOTS elements are used to design key concepts. Participants and study design are relevant elements used in all reviews. Intervention reviews require specification of the intervention’s exact nature. Outcomes are important for both intervention and prognostic reviews.

Search terms are then developed using key concepts. There are two main search terms (text words and index terms). Text words or natural language terms appear in most publications. Different authors may use different text words for the same pathology. For an example, words such as injury, wound, trauma are used to describe physical damage to the body. Index terms, on the other hand, are controlled vocabularies defined by database indexers [4]. Common terms are MeSH (Medical Subject Headings) by MEDLINE and Emtree in Embase. The index terms do not change with the interface (eg. the term ‘wound and injuries’ is used for all types of damage to the body from external causes) [5].

Search filters are used to identify search terms. The choice of filters depends on the study design, database and interface. There are specific words used to combine search terms called ‘Boolean operators’. The main Boolean operators are ‘OR’ which broaden the search (accidents OR falls will include all studies with both terms) and ‘AND’ which narrow the search (accidents AND falls will select studies with both terms). In standard search strategy all terms within a key concept are combined with ‘OR’ and in-between concepts using ‘AND’.

Limits and restrictions are used in search strategy to improve precision. The common restrictions are language selections, publication date limits and format boundaries. These limits may result in missing relevant studies. It is good practice to explain the reason for restrictions in the search strategy. It is also important to be aware of errors and retractions in selected studies. Information specialists can add terms to remove such studies in the search process. The final step is piloting the search strategy. It will give an opportunity to adjust the search strategy for optimal sensitivity and precision [6].

### Managing

All systematic reviews require consistent management of the search studies. It is challenging to manage a large number of studies manually. Reference management software can merge all search results, remove duplicates, record number of studies selected in each step, store methodology and selection criteria, and support exporting selected studies to analysis software. Specific platforms and software packages are extremely useful and can save time and effort in navigating the search and compiling the appropriate data. There are many software packages available for systematic review reference management, including Covidence, Abstracker, CADIMA, SUMARI and DistillerSR.

Throughout the search process, documentation is crucial. Search criteria and strategy, total number of studies in each step, searched databases and non-databases and copies of internet results are important records. In a situation where the search was more than 12 months old, it is advisable to re-run the search to minimize missing novel studies [2, 6].

## Selecting studies

**Fig. 3:**
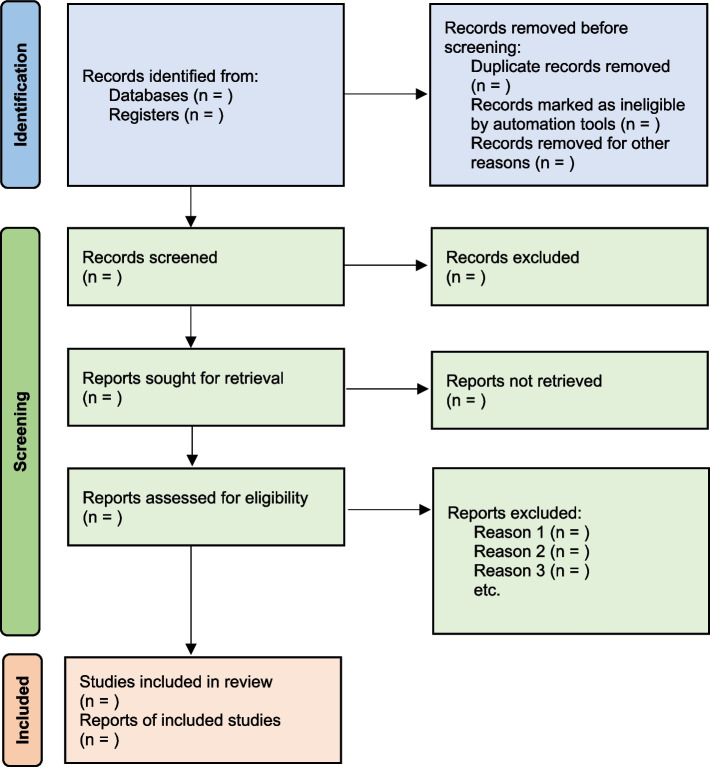
PRISMA flow diagram map for systematic review study selection process

All the searched studies are selected for quantitative synthesis. Numbers of studies marked in each selection process needs to be documented. The PRISMA flow maps (Fig. 3) can be used to report the selection process [7].

During the selection process, it is important to minimize bias. This can be achieved by measures such as having a pre-planned written review protocol with inclusion and exclusion criteria, adding study design as an inclusion criteria and independent study selection by at least 2 researchers. Items to consider in collecting data are source, eligibility, methods, outcomes, and results. Outcomes should be based on what is important to patients, not what researchers have decided to measure. Other items of interest are bibliographic information and references of other relevant studies. The most important decisions for the entire review are whether individual studies will be included or excluded for consideration in subsequent analyses. This may be the major determinant of the final composite results of the review. It is important to resolve any discrepancies in individual judgements by reviewers as objectively as possible, always remembering that individuals may be nature by “lumpers” or "splitters”. Ref (Darwin, Charles (1 August 1857). "Letter no. 2130". Darwin Correspondence Project).

Once the items to collect are decided, data extraction forms can be used to collect data for the review. The extraction form can be set up as paper, soft copy (word, excel or pdf format) or by using a database from specific software (eg: Covidence, EPPI-Reviewer, etc). All recordable outcome measures are collected for optimal analysis. It is nearly always a problem that some included studies may not provide usable data for extraction. These challenges are managed as shown in Table 2.

**Table 2:** Common challenges in selection of studies

Challenges in study selection	Acceptable solution
Different measurements	Choose the most common or valid measure Convert to a common measure Request more information from author
Different statistical analysis	Set a spreadsheet for data overview before transfer to statistical software
Incompatible results	Report them in the text as a narrative synthesis Report them in a separate table
Unclear data	Contact authors for clarification
Unusable or non-numerical data	Report in the review to avoid bias
No outcome of interest or selective reporting of outcomes	Request more information from authors Can exclude the study if specific outcome is not included in the eligibility criteria

It is important to be polite and clear when contacting authors. Imputing missing data carries a risk of error and it is best to get as much possible information from relevant authors. There are different data categories used to report outcomes in research studies. Table 3 summarizes common data types with some examples [2].

**Table 3:** Data types and formats

Data category	Description	Examples
Dichotomous	Two classes (also called binary data)	Disease or no disease Sleep or awake
Continuous	Data measured in a scale	Height, temperature
Ordinal	Categories with meaningful order	Clinical Fragility Scale Bristol stool chart
Time to event	Time taken before the outcome of interest for each participant	Time to death Time to discharge from hospital
Counts	Number of times an event happens (count) or events number during the study period (rate)	Number of falls per year

## Study quality and bias

The results will not represent accurate evidence when there is bias in a study. These poor-quality studies introduce bias into a systematic review. Risk of bias is decreased, and the study’s quality improved by clearcut randomization, outcome data on all participants (i.e. complete follow-up) and blinding (for both participant and outcome assessor) [2, 8].

The Cochrane Risk of bias tool (RoB) [9] can be used to assess risk of bias in Randomized Control Trials (RCTs). However, in Non-Randomized Studies of Interventions (NRSI), tools such as The Newcastle-Ottawa Scale [10], ROBINS-I [11], The DOWNS-Black [12] can be used to assess risk of bias. Please see bias domains in RCT and NRSI in Table 4.

**Table 4:** Risk of bias domains

Study design	Domains in a study	Risk of Bias	Bias Domain
RCT	Participant allocation	Recruitment of participants without random allocation	Bias in randomization Process
	Intervention/Treatment	Investigators aware of the allocation	Bias in deviation from interventions
NRSI	Participants	Exclusion of some eligible participants	Bias in participant selection
	Intervention	Multiple prognostic variables affect intervention Incomplete intervention information	Bias due to Confounding Bias in intervention classification
Both RCT and NRSI	Measuring outcome	Loss of participants in follow up Outcome raters not blind to interventions	Bias from missing outcome data Bias in measurement of outcomes
	Reporting	All the results are not reported	Bias from selective reporting Bias from non-reporting outcomes

*RCT* Randomized studies, *NRSI* Non-Randomized Studies of Intervention

Blinding and masking can minimize the bias secondary to deviation from intended interventions. Missing outcome data or attrition due to various issues such as participant withdrawal, loss to follow up and lost data are also common causes for bias in studies. Researchers use imputation to address missing data which could lead to over or underestimation of intervention effects. Sensitivity analysis can be conducted to investigate the effect of such assumptions. Selective reporting is another problem, and it is difficult to identify and sources such as clinical trial registries or published trial protocols can be used to minimize such discrepancies.

## Data analysis

Analysis of data is crucial in a systematic review and important aspect of this step are described below [2, 13].

### Effect measure

Outcome data for each selected study will be in different measures. It is important to select a comparable effect measure for all studies for the particular outcome to facilitate synthesis of overall effect measure. Common effect measures for dichotomous outcomes are risk ratios (RR), odds ratios (OR) and risk differences (absolute risk reduction - ARR). These measures are selected for the analysis based on their consistency, mathematical properties, and communication effect For DTA reviews sensitivity and specificity are commonly used.

The mean difference (MD) is the commonest effect measure of continuous outcome data. When interpreting MD, report as many details such as the size of the difference, nature of the outcome (good or bad), characteristics of the scale for better understanding of the results. However, studies in the review may not use the same scales and standardization of results may be required. The standardized mean difference (SMD) can be calculated in such situations if the same concept or measures are used. The SMD is expressed in units of Standard Deviation (SD). It is important to correct the direction of the scale before combining them. All outcome data should be reported along with a measure of uncertainty such as confidence interval (CI).

There are endpoints and changes from baseline data in studies. Endpoint scores are usually reported in standard deviations (SD) and change from baseline data present in MD. Although it is possible to combine two types of data, SMD calculations are inaccurate in such situations. It is also good practice to conduct sensitivity analyses to assess the acceptability of the choices made.

### Meta analysis

There are many advantages to performing a meta-analysis. It combines samples and provides more precise quantitative answers to the study objective. Study quality, comparability of data and data formats affect the output of the meta-analysis. The acceptable steps in meta-analysis are described in Table 5.

**Table 5:** Steps in meta-analysis

Steps	Description	Example
Identify comparisons	Use 2 at a time (pairwise) comparison Separate populations that can be studied within the comparison	Jogging vs running, jogging vs gym work, jogging vs dancing, for weight reduction Compare above in older people
Identify outcomes and effect measures	Select outcomes for each comparison (as per protocol) Then effect measure to report results	Weight reduction Increase in muscle mass (continuous outcome) MD or SMD
Collect data	Data collation from each selected study	
Combine results	Studies with more precise estimate should be give more weight	Variance is used to estimate the weight of the study
Choose statistical methods	Straightforward method	Inverse variance is used
	Mantel–Haenszel method (dichotomous data)	Suitable for small studies with low event rate
	Peto method	An additional option for odds close to one
	Assumptions about heterogeneity	Decision between fixed or random effect analysis
Present results	Forest plot	Displaying results in a graph with overall effect estimate at the bottom

### Heterogeneity

Variation across studies, more than expected by chance, is called heterogeneity. Although there are several types of heterogeneity such as clinical (variations in population and interventions), methodological (differences in designs and outcomes) and statistical (variable measure of effects), statistical heterogeneity is the most important type to discuss in meta-analysis [2, 14, 15].

**Fig. 4:**
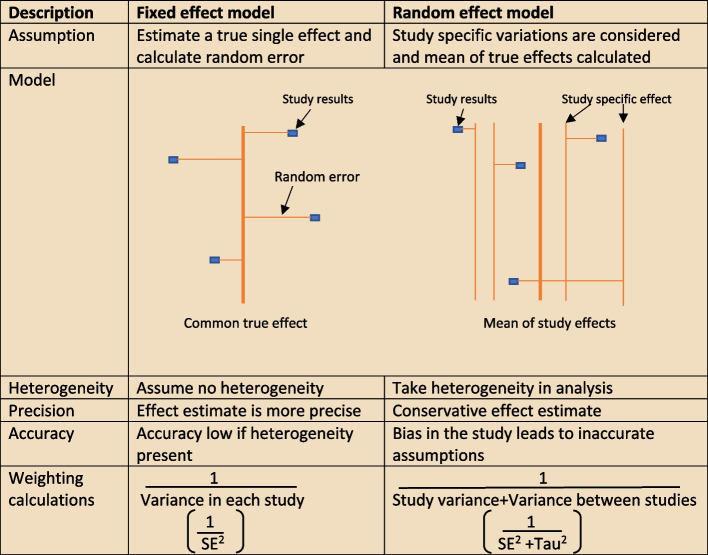
Heterogeneity assumption methods

The heterogeneity assumptions affect data analysis. There are two models as described in Fig. 4, used to assess heterogeneity. If the heterogeneity is minimal, then the Tau^2^ is close to zero and weight estimates are similar from both methods. Tau is the standard deviation of true effect between studies and Tau^2^ is the variance.

There are a few tools to assess heterogeneity. These are Q test, I^2^ statistics and visual inspection of forest plot. The easiest method is visual inspection of forest plot. Studies without overlap in confidence intervals are not homogenous. At the same time studies spread over null effect line, the heterogeneity is more relevant in analysis to guide the direction of the effect. The chi-squared or Q test believes all studies measure the same effect and a low *p* value suggests high heterogeneity. However, reliability of the Q test is low in extreme number of studies as the *p* value becomes less sensitive or too sensitive, thus under- or over-diagnosing heterogeneity respectively. The other tool to diagnose heterogeneity is I^2^ statistic, which presents heterogeneity in a percentage value. Low values, below 30%, suggest minimal heterogeneity.

The next step is to deal with heterogeneity by exploring possible causes. Errors in data collection or analysis and true variations in population or intervention are common reasons for outlying results. These identified reasons should be presented cautiously in subgroup analysis. If no cause is identified, mention this in (GRADE approach– described later) the review as unexplained heterogeneity. In each subgroup, the heterogeneity and effect modification should be reported. It is also important to have a logical basis for each factor reported in the subgroup analysis, as too many factors may confuse readers. It is equally important to make sure there is meaningful clinical relevance in these subgroups.

### Different study designs and missing data

Some studies may have more than one intervention. It is reasonable to ignore intervention arms of no interest in the review. But if all treatment arms need to be included, the control group could be divided uniformly amongst intervention arms, or all arms could be analyzed together or separately. The unit of analysis error is common in cluster randomized trial analysis, since clusters are considered as units. Similarly, correlation should be considered in crossover trials to minimize over or under weighting the study in analysis. There will be high risk of bias and heterogeneity in analyzing nonrandomized studies (NRS). However, normal effect measures can be used in relatively homogenous NRS meta-analysis.

Sometimes, missing statistics are found, and it is reasonable to calculate means and SDs from available data. Imputation of data should be done cautiously and reported in sensitive analysis.

## Reporting and interpretation of results

It is important to report results in depth and not merely statistical values. The main measures used to report meta-analysis are Confidence interval (CI) and SMD [2].

The CI is the range where the true value probably sits. A narrow CI suggests more precise effects. The CI is usually presented as 95% interval (Corresponding to *p* value of 0.05) and rarely in 90% interval (P of 0.1). It is statistically significant when CI is away from the line of zero effect. However even statistically significant effects may not have clinical value if it does not meet minimally important change. On the other effects that are not statistically significant may still have clinical importance and raises question regarding the overall power of the meta-analysis to detect clinically important effects.

The SMD is defined above (“Data analysis” section) as an effect measure. The value more than zero means significant change of the intervention. However, interpretation of the size of significance is difficult in SMD as it reports units of standard deviation (SD). The Cohen’s rule of thumb (SMD <0.4 small effect, >0.7 large effect and moderate in between), transformation to OR (assuming equal SDs in both control and intervention arms) or calculating estimate MDs in a familiar scale are reasonable methods to report SMD results.

### Reporting bias and certainty of evidence

The risk of missing information in a systematic review in the process from writing study protocol to publication is called reporting bias. Many factors such as author beliefs, word limitations, editorial and reviewers’ approvals can cause reporting bias. Funnel plots are a recommended statistical method to detect reporting bias in systematic reviews and meta-analysis.

Reporting the certainty of the results is another important step at the end of study analysis. The Grading of Recommendations, Assessment, Development and Evaluation (GRADE) is a recommended structured approach to report certainty of data. Table 6 describe topics used to rate up or down the certainty according to GRADE system [16]. Another important aspect of a systematic review is to categorize and present research studies based on the quality of the study.

**Table 6:** Rate certainty using GRADE approach

Increasing the certainty (Rate up)	Decreasing the certainty (Rate down)
• Large effect (e.g.– RR > 5) • Presence of large dose–response gradient • Opposing evidence for confounding factors (e.g. no effect showed when confounders likely to increase effect)	• Risk of bias domains- for each study and overall using RoB (2.0) tool • Inconsistency- Q test, I2 statistics and visual inspection of forest plot are used • Indirectness – Whether each study answers the review question • Imprecision- Based on information/sample size and confidence interval in overall study • Publication bias – Could use funnel plot to diagnose

The final rating of certainty in a meta-analysis is based on combination of all domains in each and overall studies. This information should be mentioned in the result section using numbers and explained in text in the discussion. The same system can be used in narrative synthesis of results in systematic reviews. It is important to remember rate up is only relevant for non-randomized studies and randomized studies starts with higher certainty.

## Reporting the review

**Fig. 5:**
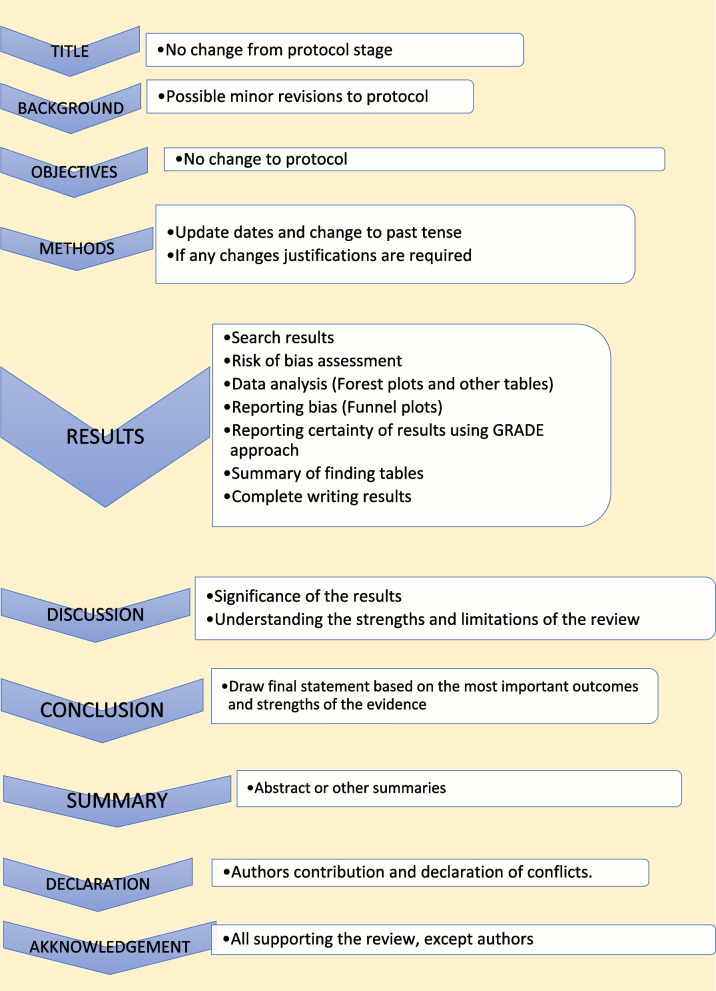
Structure for report writing

The last step of a systematic review or meta-analysis is report writing. Here, all parts are merged to write the review in structured format, using the protocol as the starting point. All systematic reviews should have a protocol to begin with as shown in Fig. 5 [2].

### Summary of finding table

The ‘summary of finding’ table is a useful step in the writing. All the outcomes with a list of studies are recorded in this table. Then the relative / absolute effect (import from forest plots), certainty of evidence (based on GRADE) and comments are included in separate columns. Footnotes can be included for explanation of decisions. There are softwares to develop summary of tables, such as GRADEpro, which is compatible with RevMan [17].

### Presenting results

The first paragraph of the results is the search process. The PRISMA flow (described in Fig. 1) is recommended to report the search summary [7]. The second section is the summary of risk of bias assessment for included studies. This will be only a narrative writing of significant differences, as individual study risk of bias will be presented in data tables in detail. Following this, review findings are presented in structured format.

The effects of interventions are presented in forest plots and data tables/figures. It is important to remember that this is not the section to interpret or infer results. All outcomes planned in the protocol should be reported, including the outcomes without evidence. Consistency of outcomes order should be maintained throughout the review. Present intervention vs no intervention before one vs other intervention. Primary outcomes are compared first, followed by secondary outcomes. Throughout the writing, check the reliability of results among plots, tables, figures, and texts. However, it may not be feasible to publish all plots and tables in the main document. Supplementary materials or appendices are available in journals for less important analyses.

There may be situations where selected studies are too diverse to conduct a meta-analysis. Narrative synthesis is an option in such situations to analyze results. It is easy to examine data by grouping studies in a narrative synthesis. Avoid vote counting of positive and negative studies in narrative reviews.

### Discussion

The first paragraph in the discussion should summarize the main (both positive and negative) findings along with certainty of evidence. The summary of the finding table can be used to identify the most important outcomes. Then describe whether the results address the study questions in the format of PICOS.

The quality of the review evidence is discussed afterwards. All domains of GRADE assessment including inconsistency, indirectness, imprecision, publication bias should be discussed in relation to the conclusions. Selection bias of studies can be included in the strengths/limitations section along with other assumptions made during the review. It is reasonable to mention agreements/disagreements with other reviews at the end in the context of past reviews.

### Conclusion

The conclusion is the summary of review findings which guide readers to make decisions in policy making or clinical practice. It is important to mention both positive and negative salient results of the review in the conclusion. Make sure only your study findings are presented, and do not comment on outside sources. At the end of presenting results, recommendations can be mentioned to fill the gaps in evidence. The primary value of systematic reviews is to drive improvements in evidence-based practice, based on the needs of patients.

### Summary

There are often other versions of the summaries from reviews presenting the major findings in plain language for the benefit of consumers and general public. It is advisable to use bullet points, and subheadings can be phrased as questions (What is the intervention? Whys it is important? What did we find? What are limitations? What is the conclusion?). It is better to write in first person active voice to directly address readers.

All types of summaries should provide consistent information to the main text. When describing uncertainty, be clear with the study limitations. As the summary is painting the study report, focus on the main results and quality of evidence.

## Data Availability

Not applicable.
